# Supplementary material for: Exploiting Differences in Heme Biosynthesis between Bacterial Species to Screen for Novel Antimicrobials
Source: Biomolecules. 2023 Oct 6;13(10):1485. doi: 10.3390/biom13101485 (PMC10604556; doi:10.3390/biom13101485)
Supplement: Supplementary file 1 [file biomolecules-13-01485-s001.zip › biomolecules-2638097-Supplementary Materials/biomolecules-2638097-supplementary-Tables S1-S2&Figures S1-S7.pdf]

Supplementary Materials

Exploiting differences in heme biosynthesis between bacterial species to screen for novel antimicrobials

Supplemental Tables:

Supplemental Table S1. Primers used to generate and validate heme gene replacements for *Sa*-CPD-YFP

Primer name	Sequence 5' to 3'
hemG_HRcas_F [1]	TGCTGGTGCTCTTTACCCCGACTTTCTGGCGTGAATGATGGAGTAATAC TCCTAATTTTTGTTGACACTCTATC
hemG_HRcas_R [1]	GGAAATTTAAAATAATTTTCTGACCGCGCATTTTTTATTCTTTATGCTTAATCAAAGGGAAAACTGTCCATATGC
SA_hY_to_hG_F [2]	TGCTGGTGCTCTTTACCCCGACTTTCTGGCGTGAATGATGGAGTAATACGTGactaaatcagtggctattataggagc
SA_hY_to_hG_R [2]	GGAAATTTAAAATAATTTTCTGACCGCGCATTTTTTATTCTTTATGCttacaactctgcgattacttcttcagc
SA_hF_cas_F [3]	TTCCACTGGTTCGACAACCAGAAAGCACATTCGAAAAAGCGATAAGTTATGTCCTAATTTTTGTTGACACTCTATC
SA_hF_cas_R [3]	AGAAGCCGTCACTGACGCTGCATCAGCGGATGCGGGAGTGGGGGTGAGGGAGTTAATCAAAGGGAAAACTGTCCATATGC
SA_hQ_to_hF_F [4]	TCCACTGGTTCGACAACCAGAAAGCACATTCGAAAAAGCGATAAGTTatgagtcaagcagccgaaacattag
SA_hQ_to_hF_R [4]	CCGTCACTGACGCTGCATCAGCGGATGCGGGAGTGGGGGTGAGGGAGttaagaaatcgcaaagaattgattgaattcg
hemoF_3F [5]	CATGGCGCAGATCTGTTTATGTCA
hemF_SahemQ_4_R [5]	CTTTCTGGTTGTCGAACCAGTGGAAATAACTGATCACGCCTTCAGCAATC
hemF_SahemQ_5_F [5]	CTGATGCAGCGTCAGTGACGGCTTCTCGGAAAACAGCTGCTGG
hemoF_6R [5]	ATCAGCCTGCAGTGCGAAAAGTG
SA_hHc_to_hH_F [6]	CTTTTCCGCTACAATTATCAACAAGTTGAATCGATAAGAGGCGGTAatgactaaaaaaatgggattattagttatggcttatgg
SA_hHc_to_hH_R [6]	CGCCGACGAGGCTCTTCGCGACGGCGCTCAGTTCTTTACCGCTCAGCTttaaaatatagacttgatttcatcaacaattgc
H_primer3 [7]	GACGTACCGGACGAACTGATCG
H_primer4 [7]	CAACTTGTTGATAATTGTAGCGGAAAAGTGAGGAAGAAGAACTAATTGCTGTGAGAG
H_primer5 [7]	GCGAAGAGCCTCGTCGGCGGTTTTCATCATCCGTGAATAATGC
H_primer6 [7]	CCACCAGCAGGCGGAGGATTATC
hemN_KO_R [8]	CTCAGCACATTGATGGCGCTTTCGTTTTCTACTTTGTAAACGAAGCGCCATTCACTATAAACAGCGAAAAAACCCCGCCCTGT
hemN_KO_F [8]	CGAAACGGGCATCCGCCGGTACGCCGTAGCCGCCAGAGACGCCATCGGAAGGAGTGAGCATAAACAAAAAAAACCCCGCTTCGGC
MC4100_hemG_F [9]	GATCCTGATTGCCAACATGCTG
MC4100_hemG_R [9]	GTGTTTGCCGTTGTTCCGTGTC
SY1_F [9]	GGATTAGAACAAGATATTGTTACAAATACGAC
SY1_R [9]	GTCGTATTTGTAACAATATCTTGTTCTAATCC
SY3_F [9]	CGTATTAGTAACGACACCACATCAAGTG
SY4_F [9]	CAGATAATGAATTAGTATCGATTGTACGTAGAG
SY2_F [9]	AGAAGAGGCATTCGGAAGTCTG
Ec_hemF_SoF [9]	GTCGGTTCCTTCGGTGCTGGTG
Ec_hemF_SoR [9]	CTGAAACGCATTCGCCTGAACG
F_primer4_part_R [9]	AATAACTGATCACGCCTTCAGCAATC
F_primer5_part_F [9]	CGGAAAACAGCTGCTGGTAATCC
MC4100_hemH_F [9]	CCTTTTTCTGATTTGACCTCTCACAGC
MC4100_hemH_R [9]	CTACCCAGGCACGCTGCACG
H_primer4_part_R [9]	GAGGAAGAAGAACTAATTGCTGTGAG

H_primer5_part_F [9]	GTTTTCATCATCCGTGAATAATGC
Ec_hemN_SoF [9]	CCGTTCTGCTGATAACCCTTCCG
Ec_hemN_SoR [9]	CATCAGGCATCATGCTCAGATGC
qPCR1_Ec_hemG_F [10]	TCTGATGAACTCGCAATGGC
qPCR1_Ec_hemG_R [10]	TTTGCGCGTATCCGTTTCAC
qPCR1_Ec_hemH_F [10]	ATGAACTGGCACGCATTCTG
qPCR1_Ec_hemH_R [10]	TTGGCAAAAGAAGCGCGTAC
qPCR1_Ec_hemF_F [10]	ATTTCGACCGCTGTTTTGCC
qPCR1_Ec_hemF_R [10]	TTTCCGTCGCTCGACAATTG
qPCR1_Ec_hemN_F [10]	TTGCACTGCTTAACCATGCG
qPCR1_Ec_hemN_R [10]	ACACGTTTCAGGGTAAAGGC
qPCR1_Sa_hemQ_F [10]	ATGAGTCAAGCAGCCGAAAC
qPCR1_Sa_hemQ_R [10]	TTCAGTGACAAGTGCATCGC
qPCR1_Sa_hemH_F [10]	GTTTCGGCACATAGTTTGCC
qPCR1_Sa_hemH_R [10]	TGCCAACCAATCGCGATATG
qPCR1_Sa_hemY_F [10]	TGCCGAAAAGTATGCCACAG
qPCR1_Sa_hemY_R [10]	CCAACCGCTTCAAAAGATGC
U16SRT-F (16S) [11]	ACTCCTACGGGAGGCAGCAGT
U16SRT-R (16S) [11]	TATTACCGCGGCTGCTGGC

[1] Replacement of *E. coli pgdH1* with *tetA-sacB* selection/counterselection cassette

[2] Replacement of *tetA-sacB* selection/counterselection cassette in *E. coli pgdH1* site with *S. aureus cgoX*

[3] Replacement of *E. coli cgdC* with *tetA-sacB* selection/counterselection cassette

[4] Replacement of *tetA-sacB* selection/counterselection cassette in *E. coli cgdC* site with *S. aureus chdC*

[5] Generation of longer integration arms for *E. coli cgdC* site (3&4 3' end, 5&6 5'end)

[6] Replacement of *E. coli ppfC* with *S. aureus cpfC*

[7] Generation of longer integration arms for *E. coli ppfC* site (3&4 3' end, 5&6 5'end)

[8] Amplification of KmR marker (flanked by FRT sites) from pKIKOlacZKn plasmid for replacement of *E. coli cgdH*

[9] Additional primers used for sequence verification of integrated DNA

[10] Primers used for qPCR analysis

Supplemental Table S2. qPCR analysis confirms gene replacements and expression. RNA was extracted and reverse transcribed from log phase and stationary phase bacteria. MC4100-YFP is the parent strain of the screen's *Sa*-CPD-YFP bacteria. cDNA was diluted 1/1000 for 16S qPCR analysis. "Ct" = cycle threshold. N=3 for 16S, all others n=1. "no Ct" = Ct > 34 cycles.

		**Log phase cultures**	**Stationary cultures**
**sample**	**primer set**	**Ct**	**Ct**
MC4100-YFP	16S	13.10	13.16
Sa-CPD-YFP	16S	13.30	13.43

MC4100-YFP	cgdC	24.43	25.68
MC4100-YFP	chdC	no Ct	no Ct
Sa-CPD-YFP	cgdC	no Ct	no Ct
Sa-CPD-YFP	chdC	22.92	27.49
MC4100-YFP	pgdH1	21.43	24.82
MC4100-YFP	cgoX	no Ct	no Ct
Sa-CPD-YFP	pgdH1	no Ct	no Ct
Sa-CPD-YFP	cgoX	21.41	26.31
MC4100-YFP	ppfC	22.32	23.95
MC4100-YFP	cpfC	no Ct	no Ct
Sa-CPD-YFP	ppfC	no Ct	no Ct
Sa-CPD-YFP	cpfC	22.07	24.07

Supplemental Figures:

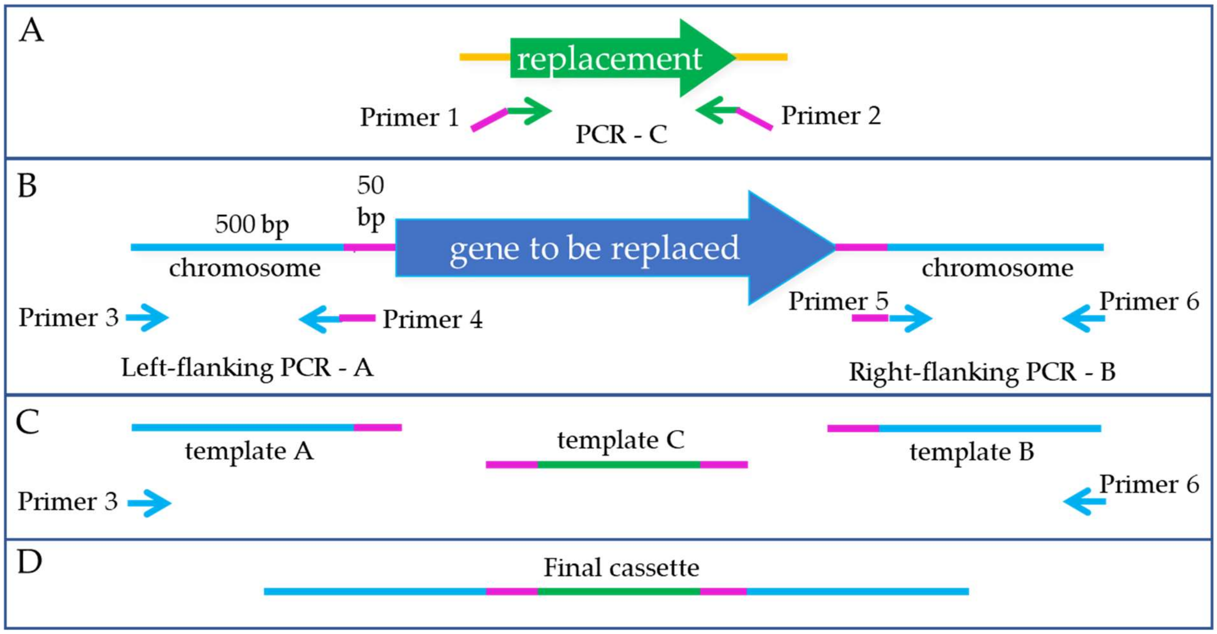

Supplemental Figure S1. Three part PCR Schematic: (A) PCR reaction amplifies replacement cassette, "PCR–C"; (B) additional adjacent regions of homology are also amplified, with some overlap, "PCR-A" & "PCR-B"; (C) PCR products A, B & C are combined as templates, which together with outer primers are used to amplify a replacement cassette with larger regions of homology (D).

Supplemental Figure S2:

***Sa*-CPD-YFP (ex/em 500/535)**

	1	2	3	4	5	6	7	8	9	10	11	12
A	0	136	132	134	115	114	114	118	104	96	121	
B	0	0	108	65	92	88	89	79	97	85	98	111
C	0	111	84	84	82	84	87	85	93	93	89	98
D	0	102	83	2	**4**	90	87	80	82	90	86	95
E	0	90	88	80	15	42	78	81	85	89	86	101
F	0	98	94	88	90	82	88	89	89	90	89	97
G	0	81	15	80	72	67	64	77	85	93	88	102
H	0	98	89	90	88	93	89	85	87	85	88	98

WT-CFP (ex/em 445/475)

	1	2	3	4	5	6	7	8	9	10	11	12
A	-2	121	115	115	102	100	99	93	99	90	117	121
B	-1	3	90	108	104	98	100	108	92	95	92	103
C	1	121	95	103	97	98	94	92	89	90	91	103
D	0	104	98	105	**149**	101	97	95	105	89	92	96
E	-1	104	97	107	122	143	96	96	98	90	94	100
F	0	95	90	99	100	95	103	86	92	94	93	94
G	2	366	65	103	103	99	111	94	93	93	93	93
H	0	92	91	90	90	89	95	91	96	94	96	91

***Sa*-CPD-YFP + hemin (ex/em 500/535)**

	1	2	3	4	5	6	7	8	9	10	11	12
A	0	90	91	90	88	92	94	100	100	-2	99	91
B	0	1	90	74	101	88	87	118	96	53	102	88
C	0	100	89	86	85	101	110	117	112	92	104	105
D	0	98	90	5	**70**	99	96	92	92	99	98	104
E	0	98	87	174	7	53	97	100	102	92	91	104
F	0	92	111	81	71	82	91	104	88	91	88	103
G	0	109	29	78	68	70	85	114	99	98	92	98
H	0	99	100	99	92	92	96	87	104	92	98	107

WT-CFP + hemin (ex/em 445/475)

	1	2	3	4	5	6	7	8	9	10	11	12
A	0	93	98	97	94	96	92	103	91	-11	102	101
B	-1	2	95	92	102	102	96	99	95	57	98	95
C	0	103	100	98	95	104	91	106	105	90	98	104
D	-1	96	98	136	**99**	105	108	112	103	92	97	102
E	1	108	94	127	149	142	96	103	106	97	96	98
F	0	100	96	97	94	89	87	95	104	91	93	97
G	1	128	83	92	98	101	94	115	102	99	98	100
H	0	107	99	91	90	93	90	87	98	95	101	103

Supplemental Figure S2. Example microplate readings expressed as percent of control, in YFP and CFP channels for duplicate plates +/- hemin. Column 1 blanks have been subtracted, and well readings have been normalized to vehicle control wells (column 12). Well 5D is a screen hit, butylcycloheptylprodiginine. Well 5D showed selective inhibition in the *Sa*-CPD-YFP that was rescued upon addition of heme. The amount of WT-CFP bacteria was increased when growth of *Sa*-CPD-YFP was inhibited.

Plate Legend

	1	2	3	4	5	6	7	8	9	10	11	12
A	media	*Sa* -CPD-YFP + WT-CFP	*Sa* -CPD-YFP + WT-CFP	*Sa* -CPD-YFP + WT-CFP	*Sa* -CPD-YFP + WT-CFP	*Sa* -CPD-YFP + WT-CFP	*Sa* -CPD-YFP + WT-CFP + spectinomycin	*Sa* -CPD-YFP + WT-CFP + spectinomycin	*Sa* -CPD-YFP + WT-CFP + spectinomycin	*Sa* -CPD-YFP + WT-CFP + spectinomycin	*Sa* -CPD-YFP + WT-CFP + spectinomycin	Sa-CPD-YFP
B	media	*Sa* -CPD-YFP + WT-CFP	*Sa* -CPD-YFP + WT-CFP	*Sa* -CPD-YFP + WT-CFP	*Sa* -CPD-YFP + WT-CFP	*Sa* -CPD-YFP + WT-CFP	*Sa* -CPD-YFP + WT-CFP + spectinomycin	*Sa* -CPD-YFP + WT-CFP + spectinomycin	*Sa* -CPD-YFP + WT-CFP + spectinomycin	*Sa* -CPD-YFP + WT-CFP + spectinomycin	*Sa* -CPD-YFP + WT-CFP + spectinomycin	Sa-CPD-YFP
C	media	*Sa* -CPD-YFP + WT-CFP	*Sa* -CPD-YFP + WT-CFP	*Sa* -CPD-YFP + WT-CFP	*Sa* -CPD-YFP + WT-CFP	*Sa* -CPD-YFP + WT-CFP	*Sa* -CPD-YFP + WT-CFP + spectinomycin	*Sa* -CPD-YFP + WT-CFP + spectinomycin	*Sa* -CPD-YFP + WT-CFP + spectinomycin	*Sa* -CPD-YFP + WT-CFP + spectinomycin	*Sa* -CPD-YFP + WT-CFP + spectinomycin	Sa-CPD-YFP
D	media	*Sa* -CPD-YFP + WT-CFP	*Sa* -CPD-YFP + WT-CFP	*Sa* -CPD-YFP + WT-CFP	*Sa* -CPD-YFP + WT-CFP	*Sa* -CPD-YFP + WT-CFP	*Sa* -CPD-YFP + WT-CFP + spectinomycin	*Sa* -CPD-YFP + WT-CFP + spectinomycin	*Sa* -CPD-YFP + WT-CFP + spectinomycin	*Sa* -CPD-YFP + WT-CFP + spectinomycin	*Sa* -CPD-YFP + WT-CFP + spectinomycin	Sa-CPD-YFP
E	media	*Sa* -CPD-YFP + WT-CFP	*Sa* -CPD-YFP + WT-CFP	*Sa* -CPD-YFP + WT-CFP	*Sa* -CPD-YFP + WT-CFP	*Sa* -CPD-YFP + WT-CFP	*Sa* -CPD-YFP + WT-CFP + spectinomycin	*Sa* -CPD-YFP + WT-CFP + spectinomycin	*Sa* -CPD-YFP + WT-CFP + spectinomycin	*Sa* -CPD-YFP + WT-CFP + spectinomycin	*Sa* -CPD-YFP + WT-CFP + spectinomycin	WT-CFP
F	media	*Sa* -CPD-YFP + WT-CFP	*Sa* -CPD-YFP + WT-CFP	*Sa* -CPD-YFP + WT-CFP	*Sa* -CPD-YFP + WT-CFP	*Sa* -CPD-YFP + WT-CFP	*Sa* -CPD-YFP + WT-CFP + spectinomycin	*Sa* -CPD-YFP + WT-CFP + spectinomycin	*Sa* -CPD-YFP + WT-CFP + spectinomycin	*Sa* -CPD-YFP + WT-CFP + spectinomycin	*Sa* -CPD-YFP + WT-CFP + spectinomycin	WT-CFP
G	media	*Sa* -CPD-YFP + WT-CFP	*Sa* -CPD-YFP + WT-CFP	*Sa* -CPD-YFP + WT-CFP	*Sa* -CPD-YFP + WT-CFP	*Sa* -CPD-YFP + WT-CFP	*Sa* -CPD-YFP + WT-CFP + spectinomycin	*Sa* -CPD-YFP + WT-CFP + spectinomycin	*Sa* -CPD-YFP + WT-CFP + spectinomycin	*Sa* -CPD-YFP + WT-CFP + spectinomycin	*Sa* -CPD-YFP + WT-CFP + spectinomycin	WT-CFP
H	media	*Sa* -CPD-YFP + WT-CFP	*Sa* -CPD-YFP + WT-CFP	*Sa* -CPD-YFP + WT-CFP	*Sa* -CPD-YFP + WT-CFP	*Sa* -CPD-YFP + WT-CFP	*Sa* -CPD-YFP + WT-CFP + spectinomycin	*Sa* -CPD-YFP + WT-CFP + spectinomycin	*Sa* -CPD-YFP + WT-CFP + spectinomycin	*Sa* -CPD-YFP + WT-CFP + spectinomycin	*Sa* -CPD-YFP + WT-CFP + spectinomycin	WT-CFP

Sa **-CPD-YFP (YFP channel, excitation 500 emission 535)** — **Z' Factor: 0.76**

	1	2	3	4	5	6	7	8	9	10	11	12
A	-12	8340	8304	7779	7647	7585	454	294	268	958	732	20448
B	14	7206	7644	7242	7257	7134	622	750	733	743	720	18044
C	0	7526	7237	7233	7268	7360	633	352	526	745	666	18104
D	3	7114	7385	7523	7594	7170	826	541	455	741	359	19248
E	0	7195	7138	7760	7669	7797	818	869	541	855	532	242
F	-8	7033	7203	6609	7238	7179	651	844	790	865	453	232
G	-2	7522	7047	7328	7753	7720	978	797	458	869	740	225
H	6	7747	7745	7539	7793	7650	882	269	518	885	405	219

WT-CFP (CFP channel, excitation 445 emission 475) — **Z' Factor: 0.79**

	1	2	3	4	5	6	7	8	9	10	11	12
A	-20	4089	3490	3371	3354	3373	279	317	316	234	264	-92
B	1	3474	3358	3407	3223	3276	308	191	205	226	208	-157
C	4	3340	3403	3289	3255	3233	276	386	295	230	231	-129
D	30	3429	3195	3189	3315	3288	183	319	330	187	343	-137
E	-22	3355	3261	3178	3256	3191	206	210	322	193	316	7922
F	7	3355	3254	3382	3193	3339	308	229	261	250	377	8325
G	-15	3224	3278	3269	3181	3145	202	208	337	317	237	8090
H	18	3403	3347	3321	3262	3165	236	367	378	276	397	8201

Supplemental Figure S3. Microplate fluorescence readings for an experiment calculating Z′ Factors, under screen conditions, are shown for both the YFP and CFP channels. Media only control readings have been subtracted. A graphical Legend illustrating plate contents is shown at the top. Media only controls are found in column 1. Control wells containing only *Sa*-CPD-YFP bacteria are in column 12 rows A-D, control wells containing WT-CFP bacteria are in column 12 rows E-H. All other wells contain a mixture of *Sa*-CPD-YFP and WT-CFP bacteria. 50 µg/mL spectinomycin has been added to wells in columns 7-11.

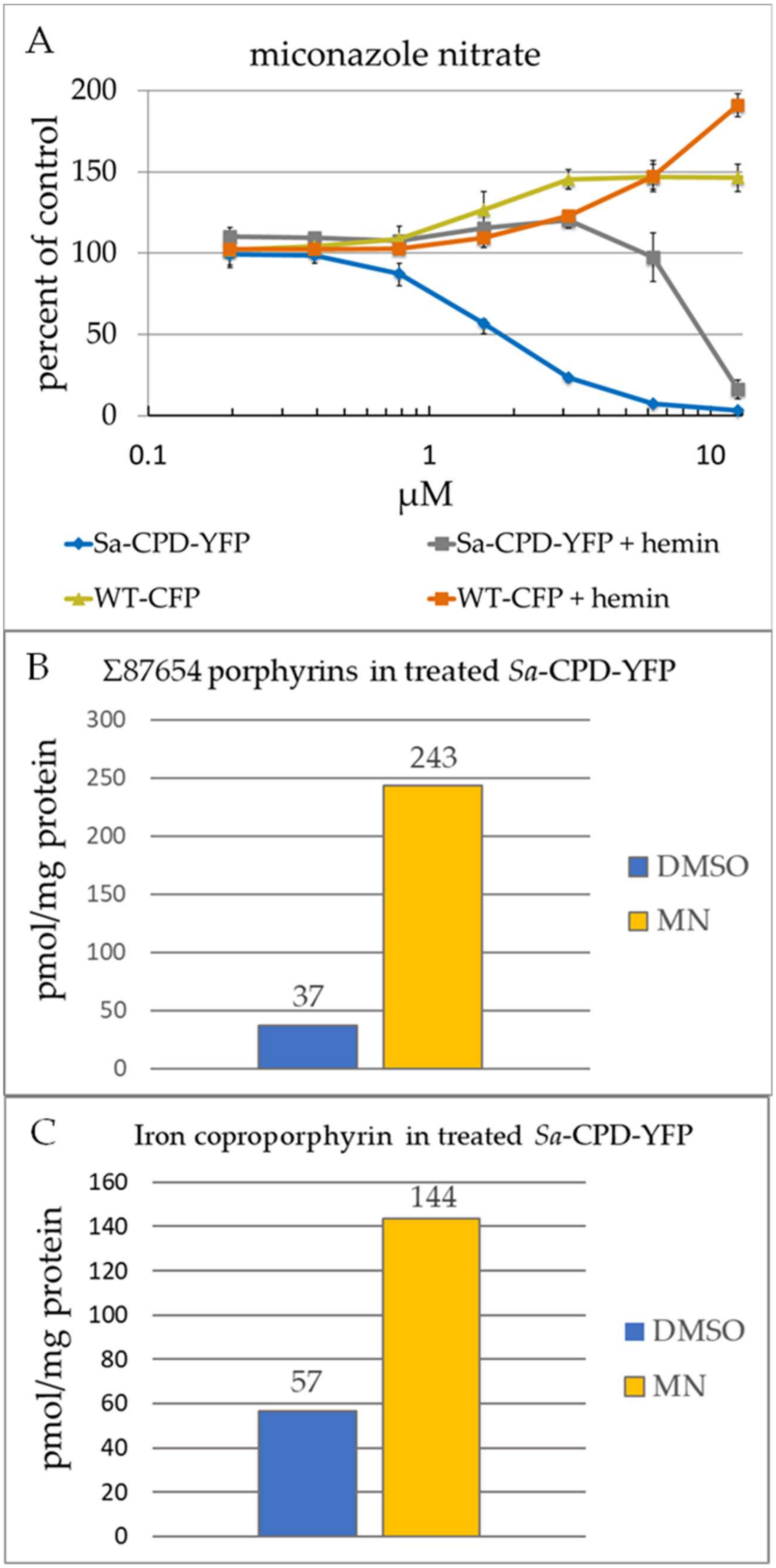

Supplemental Figure S4. (A) Serial dilutions of miconazole nitrate were used to treat mixtures of *Sa*-CPD-YFP and WT-CFP bacteria, either in the absence or presence of hemin, using assay conditions similar to those in the pilot screen. Cultures were measured at 16 hours in both YFP (*Sa*-CPD-YFP) and CFP (WT-CFP) channels. UPLC analysis was used to measure total intermediate porphyrins with 8, 7, 6, 5 & 4 carboxyl groups (Σ87654)(B) and iron coproporphyrin (C) in cell pellets from treated *Sa*-CPD-YFP bacteria. Bacteria were grown at 29 °C until O.D.$_{600}$ of 0.2, compounds were added at 6 μM and incubated for an additional 3 hours. Cells were then pelleted and washed. Porphyrins were then extracted and subjected to UPLC analysis. Preliminary analysis shows accumulation of Iron Coproporphyrin in miconazole nitrate treated *Sa*-CPD-YFP bacteria and suggests that ChdC may be inhibited in these assays. For porphyrin analysis, each sample was assayed in triplicate, sample n = 1

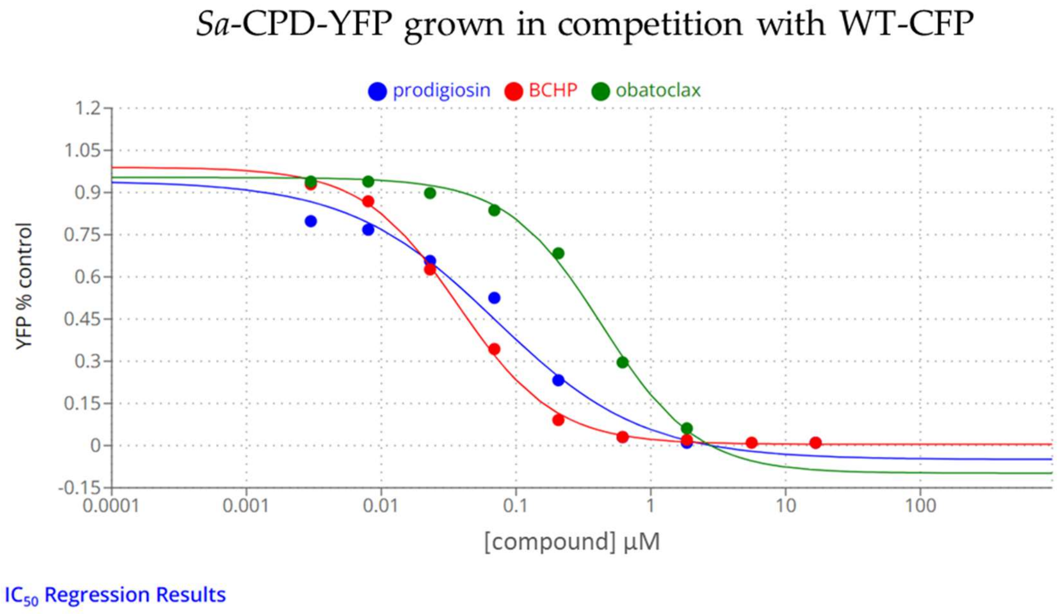

Supplemental Figure S5. Fits to determine IC50s. Mixed cultures of *Sa*-CPD-YFP and WT-CFP were cultured in the presence of prodigiosin, BCHP or obatoclax under standard screen conditions. Normalized readings from *Sa*-CPD-YFP (YFP channel) at 18 hours were plotted and IC50s calculated using an online tool: Quest Graph IC50 Calculator. Data was plotted using four parameter mode, smallest response subtracted from background correction, and divided by largest response for normalization. This analysis yielded the following IC50s: BCHP 37.4 nM, prodigiosin 70.4 nM, and obatoclax 434.6 nM. Each point on the graph is the average of four technical replicates.

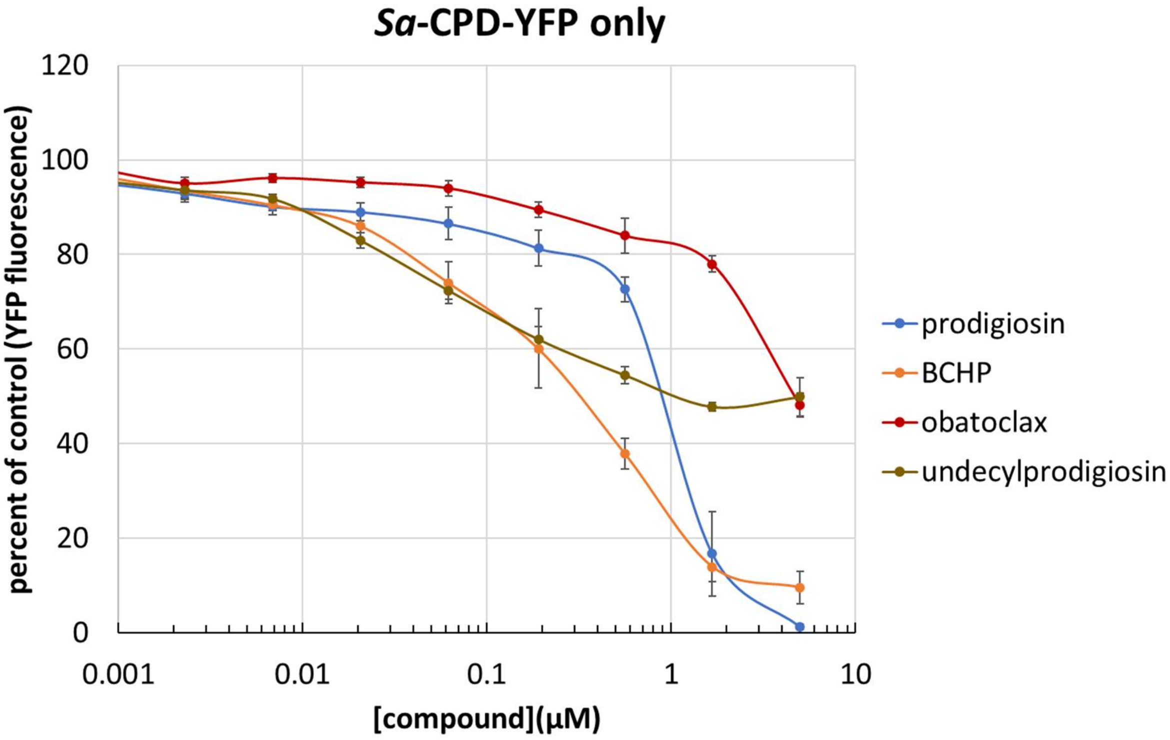

Supplemental Figure S6: A version of the screen viability assay was performed with *Sa*-CPD-YFP only (no WT-CFP). *Sa*-CPD-YFP was incubated with compound for 18 hours and *Sa*-CPD-YFP was measured in the YFP channel (excitation 500, emission 535 nm).

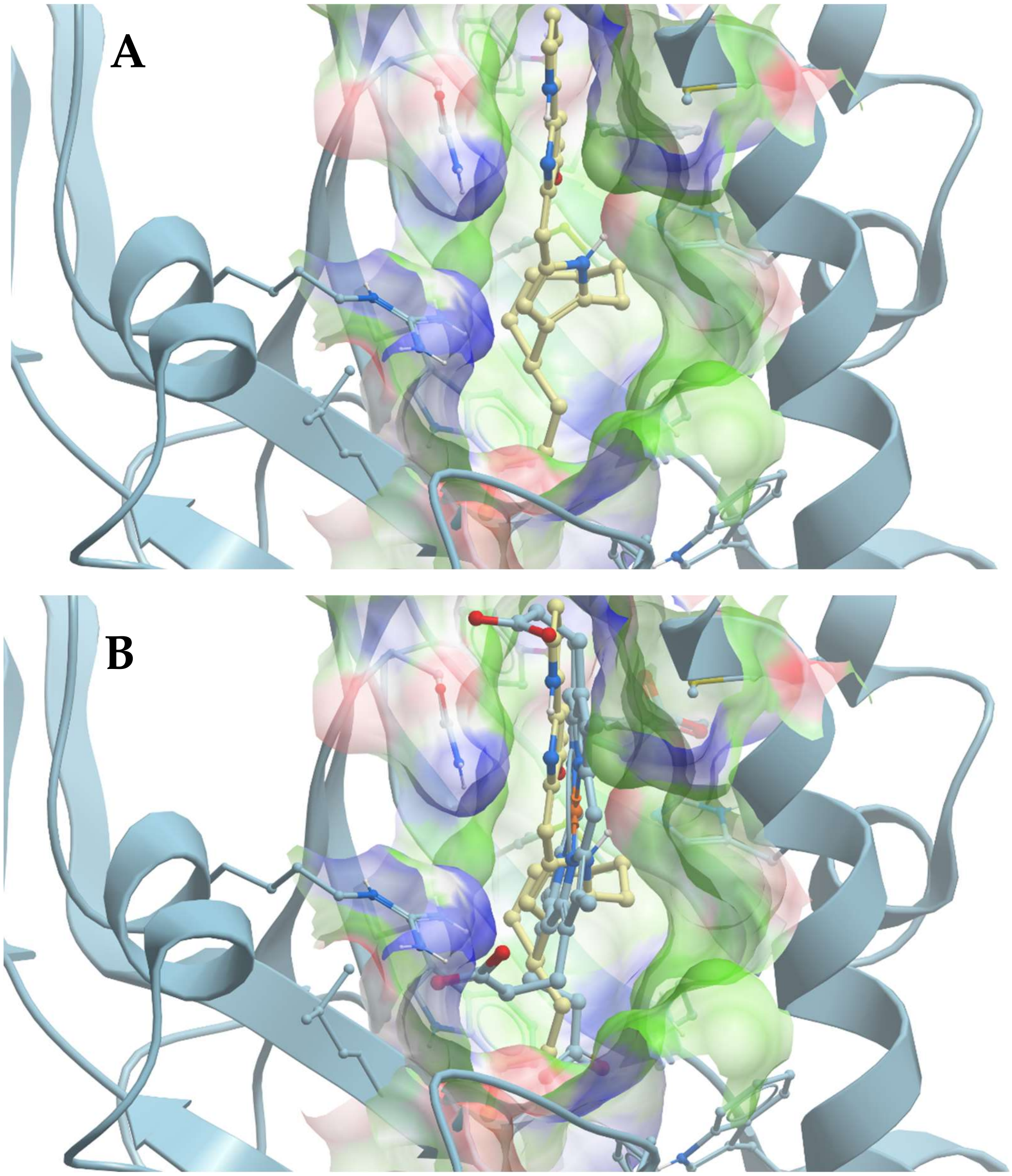

Supplemental Figure S7. Results of *in silico* docking of BCHP with LmChdC. (A) Docked BCHP is shown in the center of this figure as a thick ball and stick model (carbon yellow, oxygen red, nitrogen blue). The surrounding ligand binding pocket surface is colored by binding property (hydrophobic areas green, hydrogen bond acceptors red, and hydrogen bond donors blue). Protein is depicted by ribbon, nearby amino acid residue side chains are shown as thin ball and stick (carbons light blue) underneath the surface. (B) As in A, but coproheme (carbons light blue, thick ball and stick, Fe orange) from coproheme-LmChdC structure PDB ID: 5LOQ is shown superimposed with BCHP.
